# Efficacy of ultrasound-guided radial artery catheterization: a systematic review and meta-analysis of randomized controlled trials

**DOI:** 10.1186/cc13862

**Published:** 2014-05-08

**Authors:** Wan-Jie Gu, Hong-Tao Tie, Jing-Chen Liu, Xian-Tao Zeng

**Affiliations:** 1 Department of Anaesthesiology, the First Affiliated Hospital, Guangxi Medical University, 22 Shuangyong Road, Nanning 530021, China; 2 Department of Cardiothoracic Surgery, The First Affiliated Hospital of Chongqing Medical University, 1 Youyi Road, Chongqing 400016, China; 3 Center for Evidence-Based Medicine and Clinical Research, Taihe Hospital, Hubei University of Medicine, 32 Renmin Road, Shiyan 442000, China

## Abstract

**Introduction:**

Ultrasound guidance has emerged as an adjunct for central vein catheterization in both adults and children. However, the use of ultrasound guidance for radial arterial catheterization has not been well established. We conducted a systematic review and meta-analysis to evaluate the efficacy of ultrasound guidance for radial artery catheterization.

**Methods:**

PubMed, Embase, and the Cochrane Central Register of Controlled Trials were searched. Randomized controlled trials (RCTs) comparing ultrasound guidance with other techniques (palpation or Doppler) in adult or pediatric patients requiring radial artery catheterization were included. The primary outcome was first-attempt success.

**Results:**

Seven RCTs enrolling 546 patients met the inclusion criteria, and all the selected trials were considered as at high risk of bias. Ultrasound-guided radial artery catheterization was associated with an increased first-attempt success (relative risk (RR) 1.55, 95% confidence interval (CI) 1.02 to 2.35). There was significant heterogeneity among the studies (I^2^ = 74%). Ultrasound-guided radial artery catheterization in small children and infants also provided an increased chance for first-attempt success (RR 1.94, 95% CI 1.31 to 2.88). Ultrasound guidance further significantly reduced mean attempts to success (weighted mean difference (WMD) −1.13, 95% CI −1.58 to −0.69), mean time to success (WMD −72.97 seconds, 95% CI −134.41 to −11.52), and incidence of the complication of hematoma (RR 0.17, 95% CI 0.07 to 0.41).

**Conclusions:**

Ultrasound guidance is an effective and safe technique for radial artery catheterization, even in small children and infants. However, the results should be interpreted cautiously due to the heterogeneity among the studies.

## Introduction

Artery catheterization is a frequent and essential procedure for continuous blood pressure monitoring and arterial blood sampling in many clinical settings, including the emergency department, intensive care unit, and operating room. The radial artery is the most commonly used site for artery catheterization because of its anatomic accessibility, dual arterial supply, and the low rate of complications [1]. Traditional placement of radial artery catheterization is performed by using anatomical knowledge and pulse palpation as a guide. The estimated first-attempt success rate of radial artery catheterization with palpation differs in adult and pediatric patients, with a range from 13.8% to 68.6% [2-8]. However, the insertion of artery catheters traditionally can be challenging in small children and infants, even difficult in patients with hypotension, obesity and so on. Those special patients often require multiple attempts, which consequently cause complications, such as hemorrhage and hematoma [9]. Thus, an effective and safe alternative is urgently needed to improve radial arterial cannulation.

Ultrasound guidance has emerged as an adjunct for central vein catheterization in both adults and children. Previous studies showed the advantages of this technique in adults, including increased success rate, patient safety, and cost-effectiveness [10,11]. In children, ultrasound-guided central vein catheterization also provides significant benefits over the traditional palpation technique [12]. However, the use of ultrasound guidance for radial arterial catheterization has not been well established. Recently, several studies on the topic have been published, and the results have been conflicting [2-8]. With accumulating evidence, we therefore performed a systematic review and meta-analysis of randomized controlled trials (RCTs) to compare the efficacy of ultrasound guidance with other technique (palpation or Doppler) in adult or pediatric patients requiring radial artery catheterization.

## Materials and methods

Ethical approval and patient consent are not required since this is a systematic review and meta-analysis of previously published studies.

The systematic review and meta-analysis were conducted and reported in adherence to PRISMA (Preferred Reporting Items for Systematic Reviews and Meta-Analyses) [13].

### Search strategy and study selection

Two investigators (WJG and HTT) independently searched the following databases (inception to March 2014): PubMed, Embase, and the Cochrane Register of Controlled Trials. The electronic search strategy combined terms related to ultrasound (including a MeSH search using exp ‘Ultrasonography’, and a keyword search using the words ‘ultrasound’, ‘ultrasonography’, ‘ultrasonic’), terms related to catheterization (including a MeSH search using exp ‘Catheterization, Peripheral’, and a keyword search using the words ‘catheterization’, ‘cannulation’, ‘catheter’, ‘catheters’, ‘insertion’), and terms related to the radial artery (including a MeSH search using exp ‘Radial Artery’, and a keyword search using the words ‘radial artery’). An additional DOC file shows this in more detail (see Additional file 1). We also checked the reference lists of the screened full-text studies to identify other potentially eligible trials.

The following inclusive selection criteria were applied: (i) population: adult or pediatric patients requiring radial arterial catheterization; (ii) intervention: ultrasound-guided technique; (iii) comparison: Doppler-assisted or traditional palpation technique; (iv) outcome measure: first-attempt success; and (v) study design: RCT.

### Data extraction and outcome measures

We used a piloted data-extraction sheet, which covered the following information: first author, number of patients, population, age of patients, setting, type of control, and the experience of operators. Data were extracted independently by two investigators (WJG and HTT), and discrepancies were resolved by consensus. We contacted the corresponding author to obtain the data when necessary. Responses from authors allowed us to include one additional study in the meta-analysis [8]. No simplifications and assumptions were made. The primary outcome was first-attempt success. Secondary outcomes included mean attempts to success, mean time to success, and incidence of the complication of hematoma.

### Assessment for risk of bias and grading the quality of evidence

Assessment for risk of bias was performed in accordance with guidelines outlined in the *Cochrane handbook for systematic reviews of interventions* (version 5.1.0) [14]. Two investigators (WJG and XTZ) subjectively reviewed all studies and assigned a value of ‘high’, ‘low’, or ‘unclear’ to the following domains: random sequence generation; allocation concealment; blinding of participants and personnel; blinding of outcome assessment; incomplete outcome data; selective reporting; and other bias. Trials with high risk of bias for any one or more key domains were considered as at high risk of bias. Trials with low risk of bias for all key domains were considered as at low risk of bias. Otherwise, they were considered as unclear risk of bias [15].

The overall quality of the evidence and strength of recommendations were evaluated using GRADE [16]. GRADE Working Group grades of evidence were as follows: high quality: further research is very unlikely to change our confidence in the estimate of effect. Moderate quality: further research is likely to have an important impact on our confidence in the estimate of effect and may change the estimate. Low quality: further research is very likely to have an important impact on our confidence in the estimate of effect and is likely to change the estimate. Very low quality: we are very uncertain about the estimate.

### Statistic analysis

We estimated the relative risk (RR) with 95% confidence interval (CI) for dichotomous outcomes, and the weighted mean difference (WMD) with 95% CI for continuous outcomes. A random-effects model was used regardless of heterogeneity. Heterogeneity was reported using the I^2^ statistic, and I^2^ > 50% indicated significant heterogeneity [17]. Whenever significant heterogeneity was present, we searched for potential sources of heterogeneity. For example, if one study showed results that were completely out of range of the others, we searched for likely reasons explaining the difference and performed a sensitivity analysis excluding that study, when deemed appropriate. We further carried out subgroup analysis according to the type of insertion (elective vs. emergency). We estimated the difference between the estimates of the subgroups according to tests for interaction [18]. The *P* value <0.05 indicates that the effects of treatment differ between the tested subgroups. Potential publication bias was assessed by visually inspecting of the Begg funnel plots in which the log RRs were plotted against their standard errors (SEs). The presence of publication bias was also evaluated by using the Begg and Egger tests [19,20]. Results were considered as statistically significant for *P* <0.05. All statistical analyses were performed using Stata 12.0 (Stata Corporation, College Station, TX, USA) and RevMan 5.2 (The Nordic Cochrane Centre, Copenhagen, Denmark).

## Results

### Study selection and characteristics

**Figure 1:**
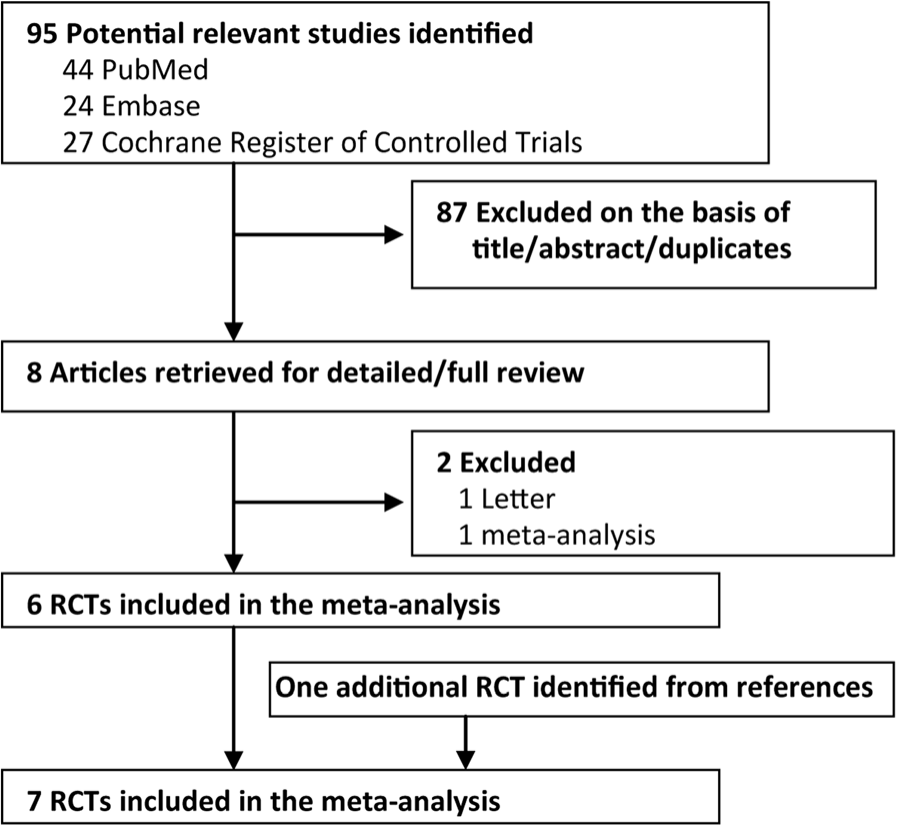
**Flowchart of the literature search and selection.** RCT, randomized controlled trial.

A detailed flowchart of the search and selection results is shown in Figure 1. Of 95 potentially relevant articles identified initially, six were included in the meta-analysis [2-7]. An additional RCT was identified from the references [8]. Finally, seven RCTs that met our inclusion criteria were included in the meta-analysis [2-8].

The main characteristics of the seven included RCTs are presented in Table 1. These studies were published between 2003 and 2013. Of the seven included studies, three were conducted in USA [4,5,7], one in Israel [2], one in Germany [3], one in France [6], and one in Japan [8]. The sample size of the RCTs ranged from 30 to 152 (a total of 546). Four studies enrolled small children and infants [3,5,7,8], and the remaining three studies included adults [2,4,6]. Six studies used the traditional palpation technique as control [2-6,8], whereas one study used the Doppler-assisted technique [7]. In three studies [2,4,8], the operators had experience of ultrasound-guided central venous catheterization but no experience of ultrasound-guided arterial catheterization; while in another three studies [3,5,7], the operators had varying degrees of experience of ultrasound-guided arterial catheterization; and in only one study [6], the operators were physicians with absence of an observational training period. Among the seven studies included here, all reported first-attempt success [2-8], two reported mean attempts to success [2,3], four reported mean time to success [2-4,8], and three reported the incidence of hematoma [4,7,8].

**Table 1:** Characteristics of the included randomized controlled trials

**Study**	**Number of patients (ultrasound/control)**	**Population**	**Age**	**Setting**	**Control**	**Operator**
Levin *et al*., 2003 [2]	69 (34/35)	Adult	Ultrasound/Palpation: 59.9 yr/66.4 yr (mean)	Elective abdominal, cardiothoracic, vascular surgery and neurosurgery	Palpation	Anesthetists with experience of ultrasound-guided central venous catheterization but no experience of ultrasound-guided arterial catheterization
Schwemmer *et al*., 2006 [3]	30 (15/15)	Small children and infants	28 months (median)	Elective neurosurgery	Palpation	Anesthetists with experience of >20 ultrasound-guided arterial catheterization
Shiver *et al*., 2006 [4]	60 (30/30)	Adult	≥18 yr	Emergency department	Palpation	Anesthetists with experience of ultrasound-guided peripheral and central venous catheterization but no experience of ultrasound-guided arterial catheterization
Ganesh *et al*., 2009 [5]	152 (72/80)	Children	Ultrasound/Palpation: 99.1 months/99.6 months (mean)	Elective abdominal, craniofacial, orthopedic, thoracic surgery and neurosurgery	Palpation	Anesthetists with experience of <10 ultrasound-guided arterial catheterization
Bobbia *et al*., 2013 [6]	72 (37/35)	Adult	Ultrasound/Palpation: 69 yr/71 yr (mean)	Emergency department	Palpation	Physicians with absence of an observational training period
Ueda *et al*., 2013 [7]	104 (52/52)	Small children and infants	Ultrasound/Doppler: 6 months/5 months (median)	Elective major surgery (mainly cardiac surgery)	Doppler	Anesthetists with experience of <5 ultrasound-guided arterial catheterization
Ishii *et al*., 2013 [8]	59 (59/59)*	Small children and infants	18.4 months (median)	Elective cardiac surgery for congenital heart disease	Palpation	Anesthetists with experience of ultrasound-guided central venous catheterization but no experience of ultrasound-guided arterial catheterization

*Matched data (the radial arteries are matched as they belong to the same patient).

### Risk of bias and grades of evidence

**Figure 2:**
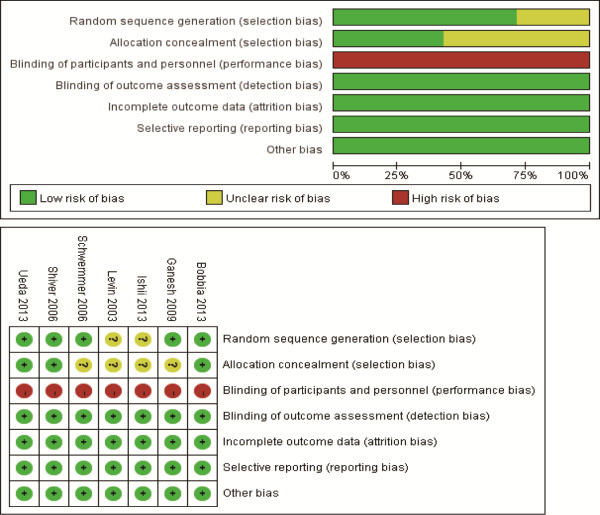
Assessment for risk of bias.

The details for risk of bias tool are shown in Figure 2. Randomized sequence generation and allocation concealment were conducted adequately in most studies. Among all the selected studies, participants and personnel were not blinded. All the selected studies were considered as at high risk of bias.

GRADE Working Group grades of evidence were moderate for first-attempt success, moderate for mean attempts to success, very low for mean time to success, and moderate for incidence of the complication of hematoma.

### Primary outcome: first-attempt success

**Figure 3:**
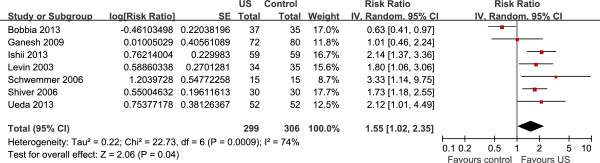
**Forest plot of first-attempt success.** US, ultrasound.

All the seven RCTs were used to calculate the pooled estimate for assessing first-attempt success [6-12]. Overall, the rate of first-attempt success in the ultrasound group and control group was 48.5% and 30.7%, respectively. Ultrasound-guided radial artery catheterization was associated with an increased first-attempt success (RR 1.55, 95% CI, 1.02 to 2.35, *P* = 0.04, Figure 3), with significant heterogeneity among the studies (I^2^ = 74%).

### Sensitivity analysis

Significant heterogeneity was observed among the included studies for the primary outcome (I^2^ = 75%). As shown in Figure 2, the study conducted by Bobbia *et al*. [6] showed results that were completely out of range of the others and probably contributed to the heterogeneity. After excluding this study, the results suggested that compared with control, ultrasound-guided radial artery catheterization was associated with an increased first-attempt success (RR 1.85, 95% CI, 1.46 to 2.32, *P* <0.00001). No evidence of heterogeneity was observed among the remaining studies (I^2^ = 0%).

### Subgroup analysis

For the primary outcome, there was no significant difference between studies of elective insertion (five trials, RR 1.91, 95% CI, 1.45 to 2.53) and studies of emergency insertion (two trials, RR 1.05, 95% CI, 0.38 to 2.83) by the test of interaction ( *P* = 0.25, I^2^ = 23.3%).

### First-attempt success in small children and infants

**Figure 4:**
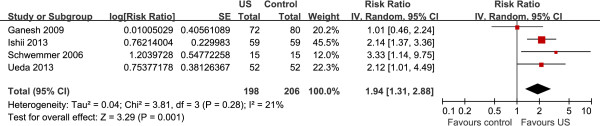
**Forest plot of first-attempt success in small children and infants.** US, ultrasound.

Figure 4 shows the pooled results from the random-effects model combining the RRs for first-attempt success in small children and infants [7,9-11]. Overall, 345 patients were included in this analysis (198 in the ultrasound group and 206 in the control group). Ultrasound-guided radial artery catheterization significantly increased first-attempt success (RR 1.94, 95% CI, 1.31 to 2.88, *P* = 0.001), with low heterogeneity among the studies (I^2^ = 21%).

### Secondary outcomes

Ultrasound-guided radial artery catheterization significantly reduced mean attempts to success (WMD −1.13, 95% CI −1.58 to −0.69, *P* <0.001), mean time to success (WMD −72.97 seconds, 95% CI −134.41 to −11.52, *P* = 0.02), and incidence of the complication of hematoma (RR 0.17, 95% CI 0.07 to 0.41, *P* <0.001).

### Publication bias

Assessment of publication bias using Egger and Begg tests showed that there was no potential publication bias among the included trials (Egger’s test, *P* = 0.58; Begg’s test, *P* = 0.30).

## Discussion

This is a further systematic review and meta-analysis of seven RCTs to evaluate the efficacy of ultrasound guidance for radial artery catheterization. The present meta-analysis suggested that compared with traditional palpation or Doppler-assisted techniques, ultrasound-guided radial artery catheterization was associated with a greater chance for first-attempt success, even in small children and infants. Additionally, ultrasound-guided radial artery catheterization significantly reduced mean attempts to success, mean time to success, and incidence of the complication of hematoma.

A previous meta-analysis on the same topic was done by Shiloh *et al*. and published in 2011 [21]. In detail, the previous meta-analysis included four RCTs for analysis, as described here [2-5], involving a total of 311 subjects, and showed that the use of ultrasound guidance for radial artery catheterization improved first-attempt success. Our meta-analysis suggested that ultrasound-guided radial artery catheterization significantly increased first-attempt success. Although consistent, the main finding of our meta-analysis generally concurs and further extends the finding of previous meta-analysis in several important ways. Our meta-analysis reinforces earlier results by including three other recently published RCTs [6-8]. These studies were high-quality and included an additional 235 patients.

We further assessed the effects of ultrasound-guided radial artery catheterization on other outcomes and found that ultrasound-guided radial artery catheterization significantly reduced mean attempts to success, mean time to success, and incidence of the complication of hematoma. The ability to reduce these outcomes provides more compelling evidence of a tangible benefit for clinicians than simply increasing first-attempt success. It is of great importance since reduced mean attempts, short mean time, and low incidence of complication give evidence that ultrasound guidance is not only effective but also expeditious and safe for radial artery catheterization.

In small children and infants, radial arterial cannulation by traditional palpation can be technically challenging, even for experienced operators, due to the small vessel diameter in pediatric patients. Our analysis suggested ultrasound-guided radial artery catheterization in small children and infants also provided a greater chance for first-attempt success when compared with traditional palpation or Doppler-assisted techniques. Interestingly, in four included pediatric studies, Ganesh *et al*. [5] differed from the other three studies and found that ultrasound guidance did not facilitate faster cannulation of the radial artery in children [9]. The negative result may be explained by: (a) the age difference of the enrolled children: children in the Ganesh study were relatively older (aged 6 to 18 yr) than those in the other three studies (small children and infants); (b) the experience level of the operators: as the authors suggested, in the Ganesh study, the operators had limited experience and lacked training; but in the other three studies, the operators, although inexperienced, had relevant experience or had received training, and were familiar with the ultrasound technique. Thus, in small children and infants, operators may need a formal demonstration on the use of the ultrasound technique using a simulated pediatric radial artery before applying it.

In some special patient populations, such as patients who are edemaous, pulseless, have anatomic variation, hypotension, obesity, and so on, the insertion of artery catheters traditionally can be particularly difficult, especially after repeated unsuccessful attempts causing complications such as hemorrhage and hematoma formation. Several case reports have confirmed that the efficacy of ultrasound-guided radial artery catheterization was even more superior in patients with anatomic variation [22], critically injured patients [23], edematous and pulseless patients [24], and hypotensive patients [25]. However, none of the studies evaluated the use of ultrasound-guided radial artery catheterization in obese patients; thus, this may be an interesting focus for future studies. Thus, one may focus on this specific patient population - namely, patients with obesity - when studying the use of ultrasound guidance for radial artery catheterization.

Although ultrasound-guided radial artery catheterization shows favorable benefits, the learning curve of ultrasound technique in radial artery catheterization may affect first-attempt success and other clinical endpoints, since ultrasound technique is a relatively new procedure and more technically difficult and complex, especially for inexperienced operators. We also believe that operators can overcome this through continued training.

This meta-analysis has several potential limitations that should be taken into account. First, our analysis is based on only seven RCTs and five of them have a modest sample size (*n* <100). Overestimation of the treatment effect is more likely in smaller trials compared with larger samples. Second, all included studies are not blinded, which may result in bias, especially in studies with a small sample size. Next, although there is no heterogeneity among the reviewed studies, patient characteristics (age difference of enrolled children), ultrasonic frequency (range from 2 to 15 MHz), and experience level of the operators differ. These factors may have a potential impact on our results. Finally, we are unable to assess the effects of ultrasound guidance on other clinically meaningful endpoints, such as patient pain, patient and physician satisfaction, because of sparse and inconsistent reporting across studies.

Further studies should focus on the following points. First, there is a need for further consistency regarding frequency of the ultrasound probe used and experience level of the operators; to date, a great variability exists in the literature. Moreover, further studies should pay more attention to clinical endpoints other than simply first-attempt success, such as patient pain, patient and physician satisfaction. Finally, none of the included studies specially evaluate the use of ultrasound guidance in difficult radial artery catheterization patients; thus, further studies should focus on the efficacy of ultrasound guidance in difficult radial artery catheterization for patients with hypotension, obesity and so on.

## Conclusions

In summary, the current available evidence suggests that ultrasound guidance is an effective and safe technique for radial artery catheterization, even in small children and infants. However, the results should be interpreted cautiously due to the heterogeneity among the studies.

## Key messages

● In adult and pediatric patients, the efficacy of ultrasound guidance for radial arterial catheterization has not been well established.

● Ultrasound-guided radial artery catheterization increases first-attempt success and further reduces mean attempts to success, mean time to success, and incidence of the complication of hematoma.

● Ultrasound guidance is an effective and safe technique for radial artery catheterization, even in small children and infants.

## Abbreviations

CI: confidence interval; PRISMA: Preferred Reporting Items for Systematic Reviews and Meta-Analyses; RCT: randomized controlled trial; RR: relative risk; WMD: weighted mean difference.

## Competing interests

The authors declare that they have no competing interests.

## Authors’ contributions

WJG conceived the study, participated in the design, collected the data, performed statistical analyses, and drafted the manuscript. HTT collected the data, performed statistical analyses, and helped to draft the manuscript. XTZ collected the data, performed statistical analyses, helped to revise the manuscript critically for important intellectual content. JCL conceived the study, participated in the design, collected the data, and revised the manuscript critically for important intellectual content. All authors read and approved the final manuscript.

## Supplementary Material

Additional file 1 **Search strategy.** This file contains the search strategy. Click here for file
