# Supplementary material for: Efficacy of ultrasound-guided radial artery catheterization: a systematic review and meta-analysis of randomized controlled trials
Source: Crit Care. 2014 May 8;18(3):R93. doi: 10.1186/cc13862 (PMC4056628; doi:10.1186/cc13862)
Supplement: Additional file 1 — Search strategy. This file contains the search strategy. [file cc13862-S1.doc]

**Additional file 1 Details of search strategy**

**PubMed**

#1 "Ultrasonography"[Mesh]

#2 "ultrasonography"[tw]

#3 "ultrasonic"[tw]

#4 "ultrasound"[tw]

#5 #1 OR #2 OR #3 OR #4

#6 "Catheterization, Peripheral"[Mesh]

#7 "catheterization"[tw]

#8 "cannulation"[tw]

#9 "catheter"[tw]

#10 "insertion"[tw]

#11 #6 OR #7 OR #8 OR #9 OR #10

#12 "Radial Artery"[Mesh]

#13 "radial artery"[tw]

#14 #12 OR #13

#15 (randomized controlled trial[pt] OR randomized controlled trials[mh] OR random allocation[mh] OR random allocat* [tw] OR randomly allo cat* [tw]

OR double-blind method[mh] OR single-blind method [mh] OR double blind* [tw] OR single blind* [tw] OR triple blind* [tw] OR clinical trial [pt] OR clinical

trials [mh]) NOT (animal [mh] NOT human [mh]))

#16 #5 AND #11 AND #14 AND #15

**Embase**

#1 'ultrasonography'/exp OR ultrasonography

#2 'ultrasound'/exp OR ultrasound

#3 'ultrasonic'/exp OR ultrasonic

#4 #1 OR #2 OR #3

#5 'catheterization'/exp OR catheterization

#6 'cannulation'/exp OR cannulation

#7 'catheter'/exp OR catheter

#8 insertion

#13 #5 OR #6 OR #7 OR #8

#14 'radial artery'/exp OR 'radial artery'

#15 #4 AND #13 AND #14

#16 #4 AND #13 AND #14 AND [randomized controlled trial]/lim AND [humans]/lim

**Cochrane Register of Controlled Trials**

#1 (ultrasound or ultrasonic or ultrasonography) and (catheterization or cannulation or catheter or insertion) and "radial artery":ti,ab,kw in Trials (Word variations have been searched)
